# Prevalence and risk factors associated with allergic rhinitis in Mexican school children: Global Asthma Network Phase I

**DOI:** 10.1016/j.waojou.2020.100492

**Published:** 2020-12-05

**Authors:** Roberto García-Almaraz, Nayely Reyes-Noriega, Blanca Estela Del-Río-Navarro, Arturo Berber, Elsy Maureen Navarrete-Rodríguez, Philippa Ellwood, Luis García Marcos Álvarez, GAN Phase I group, Valente Juan Mérida Palacio, Beatriz Del Carmen Ramos García, Alberto José Escalante Domínguez, Francisco Javier Linares Zapién, Leonardo Gardea Moreno, Georgina Guadalupe Ochoa López, Luis Octavio Hernández Mondragón, José Santos Lozano Sáenz, José Antonio Sacre Hazouri, Ma de los Ángeles Juan Pineda, Ma Guadalupe Sánchez Coronel, Noel Rodríguez Pérez, María de Jesús Ambriz Moreno, Jaime Mariano Del Río Chivardi, Omar Josué Saucedo Ramírez

**Affiliations:** a Hospital Infantil de Tamaulipas, Ciudad Victoria, Tamaulipas, Mexico; b Servicio de Alergia e Inmunología, Hospital Infantil de México Federico Gómez, Mexico City, Mexico; c Asesor Externo del Servicio de Alergia e Inmunología, Hospital Infantil de México Federico Gómez, Mexico City, Mexico; d Department of Paediatrics: Child and Youth Health, University of Auckland, Auckland 1023, New Zealand; e Pediatric Allergy and Pulmonology Units, ‘Virgen de la Arrixaca’ University Children's Hospital, University of Murcia, ARADyAL network and Biomedical Research Institute of Murcia (IMIB-Arrixaca), Murcia, Spain

**Keywords:** GAN, Allergic rhinitis, Rhinoconjunctivitis, Risk factors, Prevalence, ISAAC, International study of asthma and allergies in childhood, GAN, Global asthma network, AR, Allergic rhinitis, ARC, Allergic rhino conjunctivitis

## Abstract

**Background:**

The International Study of Asthma and Allergies in Childhood (ISAAC) showed a wide variability in prevalence and severity of allergic rhinitis (AR) and rhinoconjunctivitis (ARC), in addition to other atopic diseases (Asher et al, 2006).^1^ The Global Asthma Network (GAN) has continued to study these conditions.

**Objective:**

To estimate the prevalence of AR and ARC in children and adolescents in Mexico and to assess their association with different risk factors.

**Methods:**

GAN Phase I is a cross-sectional, multicentre survey carried out in 15 centres corresponding to 14 Mexican cities throughout 2016–2019 using the validated Spanish language version of the GAN Phase I questionnaires. The questionnaires were completed by 35 780 parents of 6–7 year old primary school pupils (children) and by 41 399 adolescents, 13–14 years old.

**Results:**

The current and cumulative prevalence of AR was higher in the adolescents (26.2–37.5%, respectively) in comparison to the children (17.9–24.9%, respectively), especially in female participants. This tendency was also observed in the current prevalence of ARC, where 15.1% of female adolescents reported nasal symptoms accompanied with itchy-watery eyes in the past year. The most important risk factors for AR and ARC were the presence of wheezing in the past 12 months, wheezing in the first year of life, the previous diagnosis of asthma and eczema symptoms. Furthermore, allergic symptoms had a negative tendency concerning altitude.

**Conclusion:**

This is the largest AR epidemiological study ever conducted in Mexico. It shows an increase in AR prevalence, as well as significant associations with modifiable risk factors, which could help to establish recommendations to reduce the burden of this condition.

## Introduction

Allergic Rhinitis (AR) is a global health problem, affecting 10–40% of the population around the world, with a prevalence of 8.38% in children and 14.93% in adolescents.^1^ It is the most common allergic disease of childhood, and its pervasiveness has increased particularly in countries with reported low prevalence values, as per the International Study of Asthma and Allergies in Childhood (ISAAC) Phase One.^1^ As a condition, AR's severity is often underestimated as it is non-life-threatening. However, the duration and severity of AR symptoms represent a substantial burden on quality of life and well-being.^2^ Crucially, AR has a detrimental effect on quality of sleep and cognitive functioning, which can cause irritability and tiredness. AR is frequently associated with comorbidities such as asthma and atopic dermatitis (AD), among others^3^

ISAAC was the first study to survey the prevalence of asthma and other allergic diseases in different countries around the world. It was carried out in 3 phases from 1992 to 2003.^1^

ISAAC Phase One was conducted from 1992 to 1995, where the prevalence of rhinoconjunctivitis (ARC) ranged between 0.8 and 14.9% (median 5.9%) in 6-7 year olds (children) and from 1.4% to 39.7% (median 13.6%) in 13-14 year olds (adolescents). In Mexico, the only centre that participated in this phase was Cuernavaca (Morelos) with an ARC prevalence of 8.6% and 9.4% in each age group, respectively. Overall, the highest prevalence rates for ARC were observed in parts of Western Europe, North America, and Australia, whereas the lowest rates were found in parts of Eastern Europe and South and Central Asia.^4^

ISAAC Phase Three (2001–2003) was conducted in centres which had participated in ISAAC Phase One, as well as other centres. The aim of the study for ARC, was to observe variations in prevalence over time, and results showed that the prevalence rates had increased in Latin America. The highest prevalence was mainly observed in centres in middle and low-income countries, particularly in Panama, where the prevalence was 39.2% in children and Brazil, with a prevalence value of 42.1% in adolescents.^5^

Ten Mexican centres participated in ISAAC Phase Three. They reported an overall prevalence of current ARC in children of 11.6% (with a range between 6.7% in Ciudad Victoria and 17.8% in Mexico City), while the adolescents reported 15.4% (with a range between 7.1% in Cuernavaca and 28.1% in Mexicali).^6^

The development of AR in the children and adolescents entails a complex interaction between genetic predisposition and environmental exposure to different factors found according to lifestyle, socioeconomic status, diet, pollution, and early development of other allergic diseases.^11^ According to ISAAC Phase Three, in Mexico, the most important risk factors for AR were the history of asthma, the presence of atopic eczema, the use of paracetamol, and history of asthma in parents.^12^^,^^13^

The aims of this study are to investigate the current prevalence of AR and ARC in children and adolescents in Mexico and to assess their association with different risk factors.

## Methods

### Study design

Global Asthma Network (GAN) Phase I is a cross-sectional, multi-centre, international, epidemiological study. Primary and secondary schools were randomly selected from a list of public and private institutions per centre to represent the target population. This phase included 15 centres in 14 cities of Mexico, including Puerto Vallarta (Vall, 7 metres above mean sea level [mamsl]), Matamoros (Mat, 8 mamsl), Mexicali (Mexi, 8 mamsl), Tijuana (Tij, 20 mamsl), Victoria City (CdVt, 316 mamsl), Cordoba (Cor, 860 mamsl), Juarez City (CdJz, 1120 mamsl), Chihuahua City (Chi, 1413 mamsl), Xalapa (Xal, 1417 mamsl), San Luis Potosi (SLP, 1864 mamsl), Aguascalientes (AgCa, 1888 mamsl), Morelia (Mor, 1920 mamsl), Mexico City (CdMx, 2250 mamsl), urban Toluca (ToUr, 2667 mamsl), and rural Toluca (ToRu, 2667 mamsl). The study was carried out in 6-7 year olds (children) where parents completed the questionnaires and 13-14 year olds (adolescents) who self-completed questionnaires at school. In both age groups, parents granted written informed consent.

### Global Asthma Network questionnaires

GAN used the same standardized written core questionnaires developed for ISAAC Phases One and Three, with the addition of doctor confirmed diagnosis of the asthma, hay fever, and eczema. In Mexico, the questionnaires were translated and back-translated from English to Spanish by 3 independent linguistic professionals, in accordance with the ISAAC English language questionnaire translation guidelines, in order to ensure that they had the same structure and logic as the original.^14^ Once the Spanish version of each questionnaire was finalized, a pilot test was carried out in schoolchildren and adolescents in Mexico City. It is worth mentioning that all the centres involved in this study applied the same version of the questionnaire by age group.

Questions were asked on demographic details such as age, sex, date of birth, school, and date of interview, as well as questions on prevalence and severity of rhinitis as well as rhinitis management and risk factors. Questionnaires were coded using a unique number for each centre, school, and participant to ensure confidentiality. In addition, height and weight measurements were taken by fieldworkers in schools.

Complete questionnaires for each age group can be consulted on http://www.globalasthmanetwork.org/surveillance/manual/study6.php and http://www.globalasthmanetwork.org/surveillance/manual/study13.php (accessed on March 23, 2020).

### Definitions

The standardized questions used in ISAAC Phase Three and GAN Phase 1 for rhinitis (hay fever) AR, ARC, and severe ARC symptoms are:

1. Have you (has your child) ever had a problem with sneezing or a runny or blocked nose when you (he or she) DID NOT have a cold or “the flu”? (PNOSEEV).
2. In the past 12 months, have you (has your child) had a problem with sneezing or a runny or blocked nose when you (he or she) DID NOT have a cold or “the flu”? (PNOSE12)
3. In the past 12 months, has this (has this child's) nose problem been accompanied by an itchy nose? (ITCH12)
4. In the past 12 months, has this (has your child's) nose problem been accompanied by itchy/watery eyes? (IEYES12)
5. In the past 12 months, how much did this nose problem interfere with your (your child's) daily activities? (Not at all, a little, a moderate amount, a lot) (IACTIV12)
6. Have you (has your child) ever had hay fever? (HFEVEREV)
7. Was your (your child's) hay fever confirmed by a doctor? (HFEVDOC)

In this study, question 1 was used to estimate the cumulative prevalence of rhinitis. Question 2 estimated AR, questions 2 and 4 were used to estimate the current prevalence of ARC symptoms (ARC12). Questions 2, 4 and the answer “A LOT” to question 5 was used to estimate the prevalence of severe rhinoconjunctivitis symptoms. Question 6 was used to estimate the prevalence of hay fever ever (also known as rhinitis ever) and question 7 was used to estimate the prevalence of rhinitis diagnosed by a doctor.^1^^,^^13^

### Sample size

A sample size of 3000 participants per age group per centre was used assuming no cluster sampling effect. The sample size provided greater than 99% (at the 1% level of significance) to detect differences in the prevalence. The average expected participation was of at least 80% for adolescents and 70% for children.^15^

### Data collection and analysis

Data were entered on an electronic database collected by the medical personnel of each study centre from August 2016 to July 2019. For quality control, 10 percent of questionnaires were double entered to mitigate possible errors. GAN databases were checked and approved in 2019, by Murcia (Spain) data centre, which was responsible for the quality control of the Spanish-and Portuguese-speaking centres. Each centre had to complete a detailed Centre Report verifying compliance with the methodological standards established by ISAAC and GAN. The report requested a description of the sampling frame, the school selection method, the number of schools excluded and rejected, the participant selection method and data entry, the record of changes made to the data, the number of children and adolescents who participated and refused, as well as a map of the sampling frame of the study area.^16^

Data analysis included central tendency measurements (mean, standard deviation [SD] and [95% CI]), as well as the cumulative and current prevalence of symptoms of rhinitis, AR, ARC, rhinitis diagnosed by a doctor, and severe ARC symptoms.

All possible factors which were likely to influence the prevalence of current and cumulative prevalence of AR or ARC were identified (p < 0.05) by Fisher and chi-squared tests. These factors were analysed by backward conditional multivariate logistic regression to create models used to conduct exploratory analysis for ARC12 risk factors. It is important to consider that although a randomization process was carried out and the sample size obtained is large in this study, the statistical method used, backward conditional multivariate logistic regression, is a method that may present a selection bias, where the correlation coefficient may be overestimated and result in an optimistic model.^48^ However, before carrying out this analysis, the candidate explanatory variables were selected according to the theoretical evidence to avoid nuisance variables and variables with a p-value <0.05 were included in the discussion, as they were the variables less prone to a selection bias. However, current validation methods such as the area under the receiver operating characteristic curve (AUC) and calibration graphs or Hosmer-Lemeshow test were not explored.^49^

Microsoft Excel 2016 v16.0.6568.2036 (Microsoft Corporation) was used to organize data and IBM SPSS Statistics v25.0 (SPSS Inc., IBM Company) was used for statistical analysis.

## Results

A total of 570 primary schools and 220 secondary schools were included during the 2016–2019 period. A total of 77 179 questionnaires were considered in this analysis. Overall, 35 780 (88.3%) children and 41 399 adolescents (91.5%) participated in the study, and the global response rate was 90%.

**Fig. 1:**
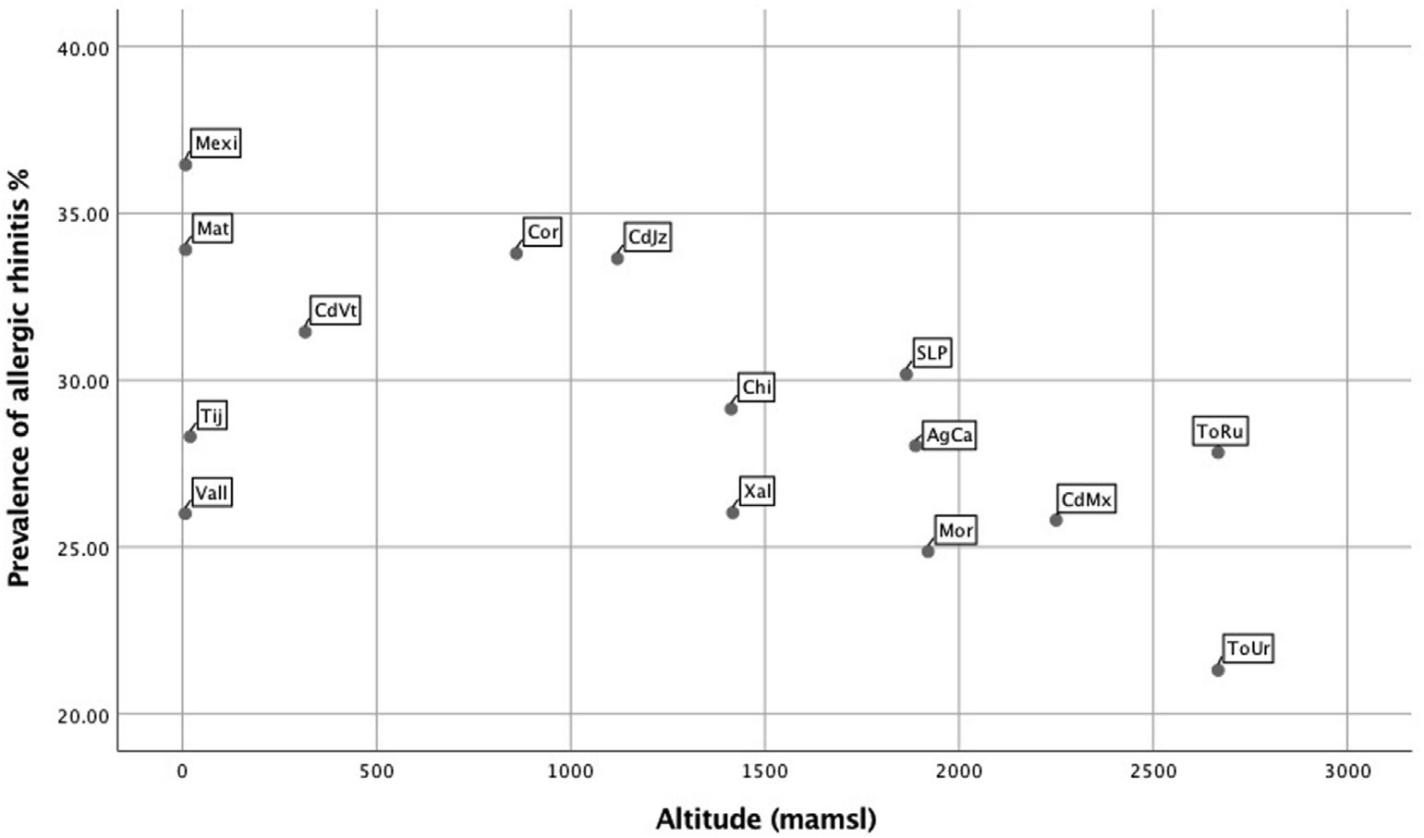
Scatter plot of prevalence values of allergic rhinitis (AR) by centre in female school children (6–7 years), according to their altitude (from the lowest to the highest)

**Fig. 2:**
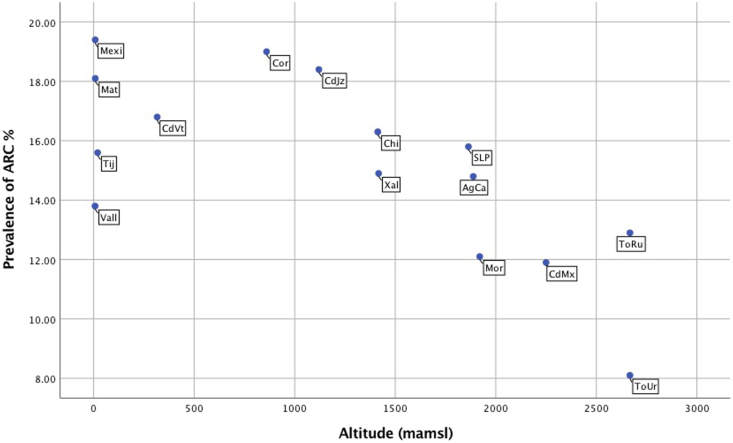
Scatter plot of prevalence values of rhinoconjunctivitis (ARC) by centre in female adolescents (13–14 years) according to their altitude (from the lowest to the highest)

The global current prevalence of AR and ARC in male and female participants are presented in Table 1 (children and adolescents). Prevalence values according to centre can be seen in Fig. 1 (children) and Fig. 2 (adolescents).

**Table 1:** Prevalence and severity of allergic rhinitis and rhinoconjunctivitis symptoms in children and adolescents from 15 centres of Mexico according to GAN 2019

Variable		Children 6–7 years old			Adolescents 13–14 years old		
		N	Frequency %	95% CI	N	Frequency %	95% CI
PNOSEEV	Males	4529/16,857	26.7	(26.1–27.5)	6712/19,456	34.4	(33.8–35.1)
	Females	4172/18,067	23.0	(22.4–23.7)	8499/21,081	40.3	(39.6–40.9)
	Total	8701/34,924	24.9	(24.4–25.3)	15,211/40,537	37.5	(37.0–37.9)
PNOSE12	Males	3325/16,921	19.6	(19.0–20.2)	4536/19,534	23.2	(22.6–23.8)
	Females	2971/18,116	16.3	(15.8–16.9)	6144/21,664	29.0	(28.4–29.6)
	Total	6296/35,038	17.9	(17.5–18.3)	10,680/40,698	26.2	(25.8–26.6)
IITCH12	Males	2121/16,910	12.5	(12.0–13.0)	2525/19,560	12.9	(12.4–13.3)
	Females	1980/18,097	10.9	(10.4–11.3)	4238/21,185	20.0	(19.4–20.5)
	Total	4101/35,007	11.7	(11.3–12.0)	6763/40,745	16.5	(16.2–16.9)
IEYES12	Males	1586/16,910	9.3	(8.9–9.8)	1953/19,554	9.9	(9.5–10.4)
	Females	1545/18,098	8.5	(8.1–8.9)	3291/2184	15.5	(15.0–16.0)
	Total	3131/35,009	8.9	(8.6–9.2)	5244/40,738	12.8	(12.5–13.1)
ARC	Males	1569/16,899	9.3	(8.8–9.7)	1878/19,489	9.6	(9.2–10.1)
	Females	1525/18,094	8.4	(8.0–8.8)	3200/21,123	15.1	(14.7–15.6)
	Total	3094/34,994	8.8	(8.5–9.1)	5079/40,616	12.5	(12.2–12.8)
HFEVEREV	Males	1590/17,114	9.2	(8.8–9.7)	1046/19,724	5.3	(4.9–5.6)
	Females	1419/18,319	7.7	(7.3–8.1)	1491/21,370	6.9	(6.6–7.3)
	Total	3009/35,433	8.4	(8.2–8.7)	2537/41,094	6.1	(5.9–6.4)
HFEVDOC	Males	1522/16,915	8.9	(8.5–9.4)	674/19,506	3.4	(3.1–3.7)
	Females	1375/18,110	7.5	(7.2–7.9)	921/21,153	4.3	(4.0–4.6)
	Total	2897/35,025	8.2	(7.9–8.5)	1595/40,659	3.9	(3.7–4.1)
IACTIV12 NONE	Males	1438/3896	36.9	(35.3–38.4)	3030/6079	49.8	(48.5–51.1)
	Females	1360/3595	37.8	(36.2–39.4)	3217/7591	42.3	(41.2–43.4)
	Total	2798/7491	37.3	(36.2–38.4)	6247/13,670	45.6	(44.8–46.5)
IACTIV12 LOW	Males	1526/3896	39.1	(37.6–40.7)	2470/6079	40.6	(39.3–41.8)
	Females	1357/3595	37.7	(36.1–39.3)	3472/7591	45.7	(44.6–46.8)
	Total	2883/7491	38.4	(37.3–39.5)	5942/13,670	43.4	(42.6–44.2)
IACTIV12 MODERATE	Males	749/3896	19.2	(17.9–20.4)	421/6079	6.9	(6.2–7.5)
	Females	722/3595	20.0	(18.7–21.3)	681/7591	8.9	(8.3–9.6)
	Total	1471/7491	19.6	(18.7–20.5)	1102/13,670	8.0	(7.6–8.5)
IACTIV12 SEVERE	Males	183/3896	4.6	(4.0–5.3)	158/6079	2.5	(2.1–2.9)
	Females	156/3595	4.3	(3.6–5.0)	221/7591	2.9	(2.5–3.2)
	Total	339/7491	4.5	(4.0–4.9)	379/13,670	2.7	(2.4–3.0)
SEVERE RHINOCONJUNTIVITIS	Males	141/15,471	0.9	(0.8–1.1)	113/17,724	0.6	(0.5–0.8)
	Females	120/16,688	0.7	(0.6–0.8)	182/18,105	1.0	(0.9–1.2)
	Total	261/32,159	0.8	(0.7–0.9)	295/35,829	0.8	(0.7–0.9)

**PNOSEEV**.- Have you (has your child) ever had a problem with sneezing or a runny or blocked nose when you (he or she) DID NOT have a cold or “the flu”? **PNOSE12**.- In the past 12 months, have you (has your child) had a problem with sneezing or a runny or blocked nose when you (he or she) DID NOT have a cold or “the flu”? **ITCH12**.- In the past 12 months, has this (has this child) nose problem been accompanied by an itchy nose? **IEYES12**.- In the past 12 months, has this (has your child's) nose problem been accompanied by itchy/watery eyes? **ARC**.- Sneezing or a runny or blocked nose when you (he or she) DID NOT have a cold or “the flu” accompanied by itchy/watery eyes**, IACTIV12**.- In the past 12 months, how much did this nose problem interfere with your (your child's) daily activities? (Not at all-none, a little-low, a moderate amount, a lot-severe), **HFEVEREV**.- Have you (has your child) ever had hay fever? **HFEVDOC**.- Was your (your child's) hay fever confirmed by a doctor? **SEVERE RHINOCONJUNTIVITIS.-** In the past 12 months, have you (has your child) had a problem with sneezing or a runny or blocked nose when you (he or she) DID NOT have a cold or “the flu”? In the past 12 months, has this (child's) nose problem been accompanied by itchy/watery eyes? and In the past 12 months, how much did this nose problem interfere with your (child's) daily activities? (a lot-severe)

The current prevalence of AR was higher in adolescents than in children (26.2% vs 17.9%) especially in female participants, where 40% reported rhinitis symptoms ever and 29% experienced AR symptoms in the past 12 months. This tendency was also observed in the current prevalence of ARC, where 15% of female adolescents reported nasal symptoms accompanied by itchy-watery eyes.

Although the adolescent group had a higher prevalence of AR and ARC symptoms, the prevalence of rhinitis diagnosed by a doctor was higher in children in comparison to adolescents (8.2% vs 3.9%, respectively).

It is important to mention that the male adolescent group reported less severe symptoms of ARC in comparison with children (0.6% vs 0.9%) and less interference on their daily activities due to nasal and ocular symptoms, contrary to the interference frequency reported by children, where it ranged from moderate (6.9%) to severe (2.5%) with a significant difference between groups (p < 0.05).

Current prevalence of AR and ARC in each centre showed great variability. Fig. 1, Fig. 2 shows the prevalence of AR and ARC rhinoconjunctivitis symptoms in different centres, according to their altitude (from the lowest to the highest). Fig. 1 corresponds to female children and Fig. 2 to the female adolescents. In female children, the cities of Mexicali and Matamoros have the highest prevalence of rhinitis symptoms (33–36%) while Morelia and Rural Toluca have the lowest ones (less than 25%). The prevalence of rhinitis confirmed by a doctor was also higher in the Mexicali and Matamoros centres.

In females of both age groups, the altitude pattern had an impact; cities under 1500 m over mean sea level (Matamoros and Mexicali) had a higher prevalence of AR while centres above 1500 m (Toluca) had the lowest prevalence. Accordingly, the centres in the cities of Matamoros and Mexicali had the highest prevalence of rhinitis confirmed by a doctor (9.5–17.7%).

Table 2, Table 3 show the risk factors for current prevalence of ARC and AR in both groups, respectively. The most important risk factors were the presence of wheezing in the past 12 months (OR 2.84 [IC95% 2.54–3.18] - 3.35 [IC95% 3.05–3.67]), wheezing in the first year of life (OR 1.48 [IC95% 1.27–1.74] - 1.77 [IC95% 1.51–2.07]), the previous diagnosis of asthma (OR 1.70 [IC95% 1.50–1.93] - 2.72 [IC95% 2.23–3.32]) and eczema symptoms (OR 1.30 [IC95% 1.06–1.58] - 2.01 [IC95% 1.62–2.49]) p < 0.001.

**Table 2:** Risk factors associated with current prevalence of rhinoconjunctivitis in children of 6–7 years and adolescents of 13–14 years old according to the Global Asthma Network (GAN) survey in Mexico

	Males 6-7		Females 6-7		Males 13-14		Females 13-14	
	OR (95%CI)	P	OR (95% CI)	p	OR (95%CI)	p	OR (95% CI)	p
Have you (has this child) ever had wheezing or whistling in the chest in the past 12 months?	3.19 (2.70–3.76)	<0.001	4.06 (3.43–4.81)	<0.001	3.32 (2.92–3.79)	<0.001	3.35 (3.03–3.70)	<0.001
Have you (has this child) ever had asthma?	2.18 (1.79–2.64)	<0.001	2.59 (2.11–3.18)	<0.001	2.05 (1.78–2.37)	<0.001	1.70 (1.50–1.93)	<0.001
Did you (this child) suffer from wheezing of whistling in the chest during his/her first year of life?	1.48 (1.27–1.74)	<0.001	1.77 (1.51–2.07)	<0.001	NI	NI	NI	NI
Have you (has this child) had this itchy rush at any time in the past 12 months?	2.92 (2.44–3.50)	<0.001	3.11 (2.59–3.72)	<0.001	2.88 (2.43–3.42)	<0.001	2.55 (2.26–2.87)	<0.001
Have you (has this child) ever had eczema?	2.01 (1.62–2.49)	<0.001	1.66 (1.33–2.06)	<0.001	1.32 (1.02–1.72)	0.033	1.56 (1.30–1.87)	<0.001
In the past 12 months, how often, on average, have you given this child/taken paracetamol for fever?								
Never	0.72 (0.57–0.91)	0.007	0.78 (0.60–1.01)	0.063	0.36 (0.30–0.42)	<0.001	0.42 (0.36–0.48)	<0.001
At least once a year	0.53 (0.45–0.61)	<0.001	0.61 (0.53–0.72)	<0.001	0.70 (0.62–0.78)	<0.001	0.64 (0.58–0.70)	<0.001
For how long was this child breastfed?					NI	NI	NI	NI
Less than 6 months	1.30 (1.09–1.56)	0.004	1.27 (1.07–1.52)	0.006	NI	NI	NI	NI
6–12 months	1.08 (0.89–1.31)	0.410	1.05 (0.87–1.26)	0.597	NI	NI	NI	NI
In the first 12 months of life, did this child have any antibiotics?	1.39 (1.19–1.62)	<0.001	1.33 (1.15–1.53)	<0.001	NI	NI	NI	NI
In the past 12 months, have you had a cat in your home?	0.99 (0.82–1.19)	0.920	0.84 (0.69–1.01)	0.075	1.16 (1.03–1.31)	0.010	1.16 (1.06–1.27)	0.001
Did you have a dog in your home during the first year of this child's life?	1.11 (0.97–1.28)	0.106	1.19 (1.04–1.36)	0.008	NI	NI	NI	NI
Altitude (mamsl) comparison <100 mts								
100–1000 m	1.25 (0.98–1.58)	0.063	0.89 (0.71–1.12)	0.333	0.77 (0.65–0.91)	0.003	1.35 (1.16–1.57)	0.000
>1000–1500 m	1.04 (0.81–1.34)	0.744	0.81 (0.62–1.04)	0.109	1.05 (0.87–1.26)	0.595	1.33 (1.12–1.57)	0.001
>1500–2000 m	1.50 (1.20–1.88)	<0.001	1.26 (1.02–1.57)	0.032	1.00 (0.84–1.20)	0.940	1.36 (1.16–1.60)	0.000
>2000–2500 m	1.23 (0.98–1.56)	0.071	1.05 (0.83–1.31)	0.668	0.87 (0.73–1.03)	0.121	1.20 (1.03–1.41)	0.019
>2500 m	1.41 (1.04–1.89)	0.024	1.16 (0.87–1.55)	0.289	0.63 (0.49–0.81)	0.000	0.99 (0.81–1.21)	0.940
NI: No information

**Table 3:** Risk factors associated with current prevalence of Allergic rhinitis in children of 6–7 years and adolescents of 13–14 years old according to the Global Asthma Network (GAN) survey in Mexico

	Males 6-7		Females 6-7		Males 13-14		Females 13-14	
	OR (95%CI)	P	OR (95% CI)	p	OR (95%CI)	p	OR (95% CI)	p
Have you (has this child) ever had wheezing or whistling in the chest in the past 12 months?	2.97 (2.58–3.42)	<0.001	3.56 (3.05–4.15)	<0.001	2.84 (2.54–3.18)	<0.001	3.35 (3.05–3.67)	<0.001
Have you (has this child) ever had asthma?	2.38 (2.00–2.84)	<0.001	2.72 (2.23–3.32)	<0.001	1.98 (1.76–2.23)	<0.001	1.83 (1.63–2.05)	<0.001
Have you (has this child) had this itchy rush at any time in the past 12 months?	2.64 (2.25–3.09)	<0.001	2.65 (2.25–3.12)	<0.001	2.34 (2.02–2.72)	<0.001	2.53 (2.27–2.83)	<0.001
Have you (has this child) ever had eczema?	1.71 (1.42–2.06)	<0.001	1.30 (1.06–1.58)	0.009	1.43 (1.15–1.78)	<0.001	1.46 (1.23–1.72)	<0.001
How often, on average did this child's mother take paracetamol in the pregnancy that she had with this child?Never	0.61 (0.25–1.46)	0.271	0.32 (0.14–0.70)	0.005	NI	NI	NI	NI
Once	0.73 (0.30–1.76)	0.494	0.38 (0.17–0.85)	0.019	NI	NI	NI	NI
Once a month	0.77 (0.31–1.87)	0.565	0.47 (0.21–1.05)	0.068	NI	NI	NI	NI
More than once a month	1.20 (0.48–3.00)	0.695	0.64 (0.28–1.48)	0.304	NI	NI	NI	NI
In the past 12 months, how often, on average, have you given this child/taken paracetamol for fever?								
Never	0.68 (0.56–0.82)	<0.001	0.73 (0.59–0.89)	0.002	0.54 (0.48–0.60)	<0.001	0.50 (0.45–0.56)	<0.001
At least once a year	0.67 (0.60–0.76)	<0.001	0.70 (0.62–0.79)	0.000	0.77 (0.70–0.84)	<0.001	0.71 (0.66–0.77)	<0.001
Was this child born prematurely (more than 3 weeks before he/she was expected)?	1.19 (0.99–1.44)	0.053	1.25 (1.08–1.46)	0.002	NI	NI	NI	NI
For how long was this child breastfed?		0.001		0.000	NI	NI	NI	NI
Less than 6 months	1.70 (1.02–1.33)	0.020	1.24 (1.08–1.43)	0.002	NI	NI	NI	NI
6–12 months	0.94 (0.82–1.08)	0.436	1.02 (0.88–1.19)	0.709	NI	NI	NI	NI
In the first 12 months of life, did this child have any antibiotics?	1.55 (1.39–1.73)	<0.001	1.44 (1.29–1.61)	<0.001	NI	NI	NI	NI
During normal week of 7 days, how many hours a day do you (does this child) spend in the following: computer and more?								
Less than 1 h/day	0.67 (0.51–0.86)	0.002	1.02 (0.70–1.48)	0.910	0.80 (0.71–0.91)	<0.001	0.74 (0.66–0.82)	0.000
One to 3 h/day	0.78 (0.60–1.02)	0.072	1.03 (0.70–1.50)	0.869	0.97 (0.88–1.07)	0.607	0.78 (0.71–0.85)	0.000
Three to 5 h/day	0.90 (0.66–1.22)	0.519	0.95 (0.62–1.45)	0.820	0.97 (0.87–1.08)	0.650	0.96 (0.87–1.05)	0.412
During normal week of 7 days, how many hours a day do you (does this child) watch television?								
Less than 1 h/day	0.86 (0.66–1.13)	0.288	0.73 (0.58–0.91)	0.007	0.81 (0.71–0.92)	0.002	0.85 (0.76–0.96)	0.008
One to 3 h/day	0.87 (0.69–1.11)	0.282	0.85 (0.69–1.04)	0.116	0.89 (0.79–1.01)	0.071	0.80 (0.72–0.89)	0.000
Three to 5 h/day	0.96 (0.75–1.24)	0.805	0.98 (0.77–1.23)	0.862	0.99 (0.87–1.12)	0.906	0.87 (0.77–0.97)	0.018
In the past 12 months, how often, on average did you (this child) eat meat (beef, lamb, chicken, pork? ComparisonNever or only occasionally								
Once or twice a week	0.74 (0.60–0.91)	0.005	0.65 (0.54–0.78)	0.000	0.76 (0.67–0.86)	0.000	0.82 (0.73–0.91)	0.001
Most or all days	0.80 (0.70–0.91)	0.001	0.68 (0.61–0.77)	0.000	0.82 (0.75–0.89)	0.000	0.82 (0.76–0.89)	0.000
Altitude (mamsl) comparison <100 mts								
100–1000 m	1.30 (1.09–1.55)	0.004	1.07 (0.90–1.28)	0.419	0.83 (0.74–0.94)	0.004	1.14 (1.02–1.29)	0.019
>1000–1500 m	1.22 (1.02–1.47)	0.028	0.97 (0.79–1.18)	0.765	0.92 (0.81–1.06)	0.284	1.10 (0.97–1.25)	0.133
>1500–2000 m	1.55 (1.31–1.83)	0.000	1.42 (1.20–1.69)	0.000	0.86 (0.76–0.98)	0.029	1.04 (0.92–1.74)	0.532
>2000–2500 m	1.32 (1.11–1.56)	0.001	1.10 (0.93–1.32)	0.249	0.88 (0.78–0.99)	0.046	1.04 (0.92–1.17)	0.474
>2500 m	1.51 (1.21–1.89)	0.000	1.34 (1.07–1.67)	0.010	0.60 (0.50–0.71)	0.000	0.93 (0.80–1.09)	0.409
NI: No information								

The use of paracetamol during pregnancy and in the past 12 months was an important factor in the children, especially in females (p < 0.05). Additionally, in the children, breast feeding duration showed a negative association with the presence of AR symptoms in both genders.

Meanwhile, in the adolescents, computer use of less than 1 h a day was a protective factor for AR (p < 0.05). Interestingly, in female adolescents, it was observed that the risk of manifesting symptoms of ARC decreased as the altitude increased. (p < 0.05).

## Discussion

This present cross-sectional study represents the most exhaustive effort to investigate the epidemiology and risk factors of AR and ARC in Mexico using a GAN methodology. Even though 32 states comprise Mexico, we consider that obtaining data from 10 states allows us to estimate the prevalence of symptoms of this disease, without the intention of assuming that it is representative of the entire country.

The cumulative prevalence of rhinitis decreased in the children (by 3.0% points) and increased in adolescents (by 4.2% points) taking ISAAC Phase Three as reference. Nevertheless, the current prevalence of nasal symptoms was lower than reported by ISAAC Phase three by 6.3% in children and 7.9% in adolescents. Similarly, the current prevalence of ARC, decreased by 0.4% in children and by 5.9% in adolescents compared to ISAAC Phase Three results.^13^

As in ISAAC Phase Three, we found wide variations among the participating centres. In children, the range of prevalence for AR was 6.6%–24.9%, and in adolescents from 12.7% −25.6% even in very close centres. Besides the worldwide ISAAC Phase Three study,^6^ many studies have been carried out during the last 20 years in children and adolescents in different Mexican cities and reported variations in the prevalence of AR and ARC in both age groups. Some of them used the ISAAC questionnaire, while others employed different validated questionnaires for AR in different age groups. For instance, Bäcker found a current prevalence of ARC of 10.5% in children in Mexicali, Baja California,^8^ and Bedolla-Barajas recorded a current prevalence of AR of 5.5% in children and adolescents aged 6 to 12 in Ciudad Guzmán, Jalisco.^9^ Furthermore, Ramirez-Soto has recently reported a global prevalence of AR of 5% and ARC of 19.2% in children in 5 cities in the central-western region of Mexico.^10^ Compared with previous Mexican results, our study reported a current prevalence of AR greater than 5 percentage points in both age groups. However, the prevalence of ARC increased only in the adolescent group.

These regional variations are attributed to diversity and difference, population size, environmental factors such as humidity or pollution and socio-economic conditions, as reported by Arnedo et al in children of different regions of Spain.^17^

Our results are consistent with previous research on the differences in prevalence according to sex. Overall AR and ARC prevalence were higher in males in comparison to females in children. This trend changes during puberty and adolescence, with female adolescents showing higher prevalence rates in comparison to males. Differences in condition prevalence is explained by higher levels of endogenous sex steroids hormones with increased Th2 response in women, whereas in men, testosterone works by suppressing the Th2 response.^18^^,^^19^ However, it has been reported that this tendency decreases during middle-age, where males have a higher prevalence of rhinitis.^20^

Several studies have shown a lower prevalence of ARC in children raised in rural environments (mainly during the first 5 years of life) and contact from birth with domestic animals. It has been proposed that these factors increase the exposure to bacterial endotoxins and high microbial diversity capable of inducing immune tolerance through Th1 stimulation and Th2 suppression, preventing the development of allergic diseases.^21^^,^^22^ Taking the aforementioned information into account, we can explain why rural centres in Toluca had the lowest prevalence of AR and ARC in children and adolescents.

This study showed an increase in the prevalence of severe symptoms of ARC in children and adolescents compared with ISAAC Phase Three (4.5% vs 0.8% and 2.7% vs 0.9%, respectively).^6^ Nevertheless, our results should not be taken as a real reflection of symptom severity, since our study only explored the level of affliction in daily life in parameters from null to severe, and did not include the criteria proposed by the Allergic Rhinitis and its Impact on Asthma (ARIA). This document proposed a system for assessing AR severity on the basis of the presence or absence of impairment in any of 4 health-related quality of life (HRQL) items: sleep, daily activities/sport, work/school, and troublesome symptoms, to classify severe rhinitis you must answer yes 3 or 4 items.^23^

For the diagnosis of rhinitis, the current prevalence of ARC and the prevalence of hay fever ever (also known as rhinitis ever) were similar in children. However, in the adolescents, less than half of the patients with symptoms of ARC and hay fever had a medical diagnosis. These data agree with the study carried out by Esteban et al where an insufficient diagnosis was reported in 24% of children with rhinitis symptoms and 53% of patients older than 7 years with AR symptoms.^24^ In Mexico, Villareal reported an AR prevalence diagnosed by a doctor of 6.7% in children of aged 6–8 and 5.4% in adolescents aged 11–14 in Ciudad Juarez, Chihuahua.^7^ One possible explanation could be that adolescents reported less deterioration in their quality of life compared to children, so they may not perceive it as a disease that requires medical attention. On the other hand, it is widely known that the diagnosis and treatment of allergic diseases depend largely on the education of the first-contact doctor and family members to detect symptoms in their mild to moderate manifestation and avoid a negative impact on patient's quality of life.^25^

AR and asthma can be unified by the concept of a “united airway,” where allergic symptoms of the upper and lower airways can be thought of as manifestations of a common atopy, where over 80% of asthmatic patients have AR^25^ and a risk factor to develop asthma is AR.^12^^,^^26^ We found in our study that asthma and AR are comorbid diseases that co-existed in 25% of the patients. Asthma also increased at least twice the risk of having AR in children and adolescents. This confirms the close correlation between AR and asthma from an epidemiological perspective.

Other risk factors for ARC and AR with greater strength and importance of association were the presence of atopic dermatitis symptoms in the past and itchy rash in the past 12 months. Overall, these factors increase twice the risk of having nasal and ocular symptoms. The ISAAC Phase Three study in Mexico reported the same risk factors in all age groups.^12^ These conditions, far from being considered as isolated conditions, must be regarded as specific manifestations of systemic allergic disease in different organs, where they can coexist by having a common allergic basis.^27^

Another risk factor with a slight association for AR and other allergic diseases was the use of paracetamol during pregnancy and in the past 12 months, according to ISAAC Phase Three in children and adolescents.^28^^,^^29^ According to our results, there was an association between the frequency of paracetamol use and the presence of AR and ARC in both age groups. In a prospective pregnancy cohort and throughout the first 6 months of life, it was observed that the consumption of paracetamol during the first trimester was associated with an increased risk of AR until 10 years of age.^30^

The Epidemiology of Allergic Diseases in Poland (EACP), a large questionnaire-based survey in the East-Central part of Europe reported that the use of paracetamol in the past 12 months was associated with a significant dose-dependent increase in the risk for developing rhinitis symptoms, with a strong correlation, in terms of the odds ratio, with the use of paracetamol at least once a month in adolescents.^31^ In line with the above, our results reported that the low consumption of paracetamol in the past 12 months was a protective factor for ARC in both age groups. The mechanism involved implies oxidative stress in the airways related to a glutathione depletion that favours inadequate protection of the respiratory mucosa with antioxidants and detectable concentrations of metabolite NAPQI in the lungs, which stimulated the transient receptor potential ankyrin-1 (TRPA1) leading to neurogenic airway inflammation.^32^^,^^33^

Breastfeeding is strongly recommended for its numerous benefits to newborns. Some studies have shown a protective effect related to time and exclusivity, while others have not only found no benefit but an increased risk of allergic diseases.^34^ A systematic review evaluated the association between exclusive breastfeeding during the first 3 months after birth and AR. It found that although breastfeeding has a protective effect, its statistical significance was borderline (OR 0.74 [95%CI 0.54–1.01]).^35^ Likewise, the PROBIT study, a large clinical trial group that used the ISAAC questionnaire, did not observe a decrease in risk at 6.5 years of age, despite the duration and exclusivity of the maternal breastfeeding.^36^ In our study, we found that breastfeeding duration lower than 6 months was significantly associated with a higher risk of AR and ARC in children.

The western pattern diet is characterized by the high consumption of red and processed meats and low consumption of vegetables and cereals. This diet is rich in polyunsaturated fatty acids and contains high levels of omega-6 fatty acids compared to omega-3, which is considered an allergy risk factor. Research has documented a higher risk of ARC and AR in children that consume animal fats 3 or more times per week compared to children who consumed animal fats once or twice a week as well as a negative association between consumption of starchy foods, rice, nuts, shellfish, and all fresh/frozen fish with the presence of symptoms of ARC, atopic dermatitis, and severe asthma in adolescents.^37^^,^^38^ However, this factor did not behave as a risk factor in our study sample, despite the high consumption of red meat.

A modern urban lifestyle implies that children and adolescents spend most of their time indoors, watching TV or playing on a computer resulting in reduced physical activity, with a potential of junk food consumption. These unhealthy lifestyle behaviours are strongly associated with the presence of allergic diseases. Computer use is a risk factor for developing AR, especially when it is used more than 3 h per day and is not frequently cleaned. This is due to computer hardware being a source of dust, hair, and mites, all of which increases the risk of contact with allergens and allergic sensitization.^39^ According to our results, this was also reported in the adolescent group where the use of computer or television for less than an hour, behaved as a protective factor for AR.

Finally, a negative tendency was found between a higher altitude (>1500 m above sea level) and a lower prevalence of ARC and AR in female adolescents. Elevated altitude (>1500 m) is thought to be an important factor in determining the incidence of asthma. As altitude increases, lower rates of asthma have been recorded.^40^ The hypothesis regarding altitude is that it significantly reduces the level of exhaled nitric oxide (NO), a determinant of inflammation of the local airways in patients with moderate or severe intrinsic asthma; increases blood levels of interleukin 10 (a cytokine with powerful anti-inflammatory properties) and decreases the concentration of interferon-γ (IFN-γ), responsible for inflammation of the local airways. Also, at higher altitudes, there is a lower concentration of allergens (pollens and mites) and air pollution.^41^^,^^42^ These observations have been reported in asthma, however, given the results per centre of AR, we consider that these observations can be extrapolated to these comorbidities.

In addition to the relationship between asthma and altitude, other studies have reported hormonal changes in females living at high altitude. For example, González and Ortiz et al reported a delay in puberty as altitude increased.^43^ Similarly to the above, it has been reported that oxidative stress due to hypoxia caused by altitude, results in late menarche, a prolonged ovarian cycle due to high levels of the hormone FSH and, therefore, a delay reproductive age in adolescents. As described above, female sex hormones promote a Th2-type inflammatory response during adolescence.^44^, ^45^, ^46^ However, based on the aforementioned results, altitude appears to have an important effect on the hypothalamic-pituitary-gonadal axis by increasing the corticotropin-releasing hormone and negatively affecting the hypothalamic secretion of gonadotropin-releasing hormone, thus reducing the release of gonadotropins.^47^

In accordance with our results, it was observed that the prevalence of AR and ARC was higher in females belonging to the centres with lower altitude above sea level compared to males. It is possible that the females who participated in the height centres (more than 1500 mamsl) such as Toluca could present low levels of sex hormones and therefore, a lower inflammatory response of the Th2 type during adolescence. However, there is no record of gynecological history or any biochemical marker that allows us to assert this argument since the questionnaire used did not address these biological aspects of the patients, therefore, no conclusions can be drawn regarding this effect in the female sex yet.

### Limitations

This multicentre cross-sectional study has limitations typical of an observational study. Apart from questionnaire responses, we didn't apply any objective measure or clinical evaluation to confirm rhinitis symptoms or diagnosis in these populations. On the other hand, self-reporting of symptoms in the adolescent group could lead to higher estimates on the presence of AR and ARC symptoms and the self-selection of centres included in this study might not be representative of the country. However, GAN has shown that this type of study is an adequate and high quality method to explore the prevalence of symptoms related to allergic diseases, which have been increasing in the last decade internationally. The information was obtained with a common methodology with a previously validated instrument and with a high response rate from all the centres involved. This study allows us to identify important associations and potential risk factors by sex and age. Additionally, environmental factors such as altitude can be established as a possible modulating factor for current wheezing in the Mexican population. This opens up the opportunity to carry out prospective studies to analyse modifiable environmental factors related to the increased prevalence of AR and ARC in different areas of the world. It represents an area of opportunity to develop detection strategies for the population at risk with symptoms of rhinitis with allergic comorbidities.

## Conclusion

This is the first report of the largest epidemiological study that has been carried out in Mexico related to AR and ARC in children and adolescents. It gives us a global idea of the magnitude of this health problem in the country, showing an increasing trend throughout the last 18 years, especially in cities under 1500 m over mean sea level.

This study allows us to confirm the association between allergic diseases and other risk factors previously reported and describe modifiable factors such as consumption of paracetamol in pregnancy, the duration of the breastfeeding and television and computer use for long periods of time. Although we are far from establishing causal relationships, we can give preventive recommendations to avoid the impact of this disease.

## GAN Phase I group

**Mérida Palacio Valente Juan**- Baja California, Mexico. MD, drvalente@clinicadeasma.com, **Ramos García Beatriz Del Carmen**- San Luis Potosí, Mexico. MD, b.aty@live.com.mx, **Escalante Domínguez Alberto José**- Hospital General Tijuana, Baja California, Mexico. MD, drajed@yahoo.com.mx, **Linares Zapién Francisco Javier**- Toluca, Mexico. MD, fjlinaresz@prodigy.net.mx, **Gardea Moreno Leonardo**- Chihuahua, Mexico. MD, hmorenogardea49@gmail.com, **Ochoa López Georgina** - Ciudad Juárez, Chihuahua. Mexico. MD, gina8a_77@hotmail.com, **Hernández Mondragón Luis Octavio**- Morelia, Michoacán, Mexico. MD, dr.hdezmondragon@gmail.com, **Lozano Sáenz José Santos**- Xalapa, Veracruz, Mexico. MD, Lozanosaenz57@gmail.com, **Sacre Hazouri José Antonio**- Córdoba, Veracruz, Mexico. MD, sacre_1@hotmail.com, **Juan Pineda Ángeles**- Puerto Vallarta, Jalisco, Mexico. MD, angjuan@hotmail.com, **Sánchez Coronel Ma. Guadalupe**- Aguascalientes, Mexico. MD, mgsc4@hotmail.com, **Rodríguez Pérez Noel**- Matamoros, Tamaulipas, Mexico. MD, drnoelrodriguez@me.com, **Ambriz Moreno María de Jesús**, Tamaulipas, Mexico. MD, draambriz@latinmail.com, **Del Río Chivardi Jaime Mariano**- Mexico City, Mexico. MD, delriojaime@yahoo.com, **Saucedo Ramírez Omar Josué**- Mexico City, Mexico. MD, dr.omar.saucedo@gmail.com.

## Authors' contributions

**GAR-** Made substantial contributions to conception and drafting the manuscript.

**RNN.-** Made substantial contributions to design, acquisition of data and drafting the manuscript.

**DRNBE.-** Made substantial contributions to conception, design, acquisition of data and drafting the manuscript.

**BA.-** Made substantial contributions to the analysis, interpretation of data and drafting the manuscript.

**NREM.-** Made substantial contributions to design and acquisition of data.

**EP.-** Made substantial contributions to the analysis and drafting the manuscript.

**GMAL.-** Made substantial contributions to the analysis and drafting the manuscript.

**MPV.-** As centre coordinator, made substantial contributions on the acquisition of data.

**RGBDC.-** As centre coordinator, made substantial contributions on the acquisition of data.

**EDAJ.-** As centre coordinator, made substantial contributions on the acquisition of data.

**LZF.-** As centre coordinator, made substantial contributions on the acquisition of data.

**GML.-** As centre coordinator, made substantial contributions on the acquisition of data.

**OLGG.-** As centre coordinator, made substantial contributions on the acquisition of data.

**HMLO**.- As centre coordinator, made substantial contributions on the acquisition of data.

**LSJS.-** As centre coordinator, made substantial contributions on the acquisition of data.

**SHJA.-** As centre coordinator, made substantial contributions on the acquisition of data.

**JPMA.-** As centre coordinator, made substantial contributions on the acquisition of data.

**SCMG.-** As centre coordinator, made substantial contributions on the acquisition of data.

**RPN.-** As centre coordinator, made substantial contributions on the acquisition of data.

**AMMDJ.-** As centre coordinator, made substantial contributions on the acquisition of data.

**DRNBE.-** Involved in revising it critically for important intellectual content.

**SROJ.-** Involved in revising it critically for important intellectual content.

## Availability of data and materials

The datasets used and/or analysed during the current study are available from the corresponding author on reasonable request.

## Ethics approval and consent to participate

The authors declare that all procedures were carried out in accordance with the ethical standards of the institutional committee on human investigation, the World Medical Association, and the Helsinki Declaration.

The authors obtained informed consent from the parents or guardians of participants in the present study. The corresponding author accepts responsibility for this manuscript.

The present study was approved by the Ethics, Research, and Biosafety committees of the Hospital Infantil de México Federico Gómez (HIMFG, protocol HIM/2016/065) in accordance with the guidelines of the institution.

Authors' consent for publication. If any of this information is not applicable, we ask that you please provide a statement to this effect.

## Funding

No financial support for this work that could have influenced its outcome.

## Agreement to publish the work

All authors consent to the publication of this work.

## Declaration of competing interest

The authors declare that they have no conflict of interest in relation to the methods or materials employed in this study.
